# The WHO Tobacco Convention: A New Dawn in the Implementation of International Health Instrument? Comment on "The Legal Strength of International Health Instruments - What It Brings to Global Health Governance?"

**DOI:** 10.15171/ijhpm.2017.70

**Published:** 2017-07-05

**Authors:** Ebenezer Durojaye

**Affiliations:** Dullah Omar Institute, University of Western Cape, Cape Town, South Africa.

**Keywords:** Tobacco Convention, Global Public Health, International Law, Implementation, National Level

## Abstract

The Tobacco Convention was adopted by the World Health Organization (WHO) in 2003. Nikogosian and Kickbusch examine the five potential impacts of the Tobacco Convention and its Protocol on public health. These include the adoption of the Convention would seem to unlock the treaty-making powers of WHO; the impact of the Convention in the global health architecture has been phenomenal globally; the Convention has facilitated the adoption of further instruments to strengthen its implementation at the national level; the Convention has led to the adoption of appropriate legal framework to combat the use of tobacco at the national level and that the impact of the Convention would seem to go beyond public health but has also led to the adoption of the Protocol to Eliminate Illicit Trade in Tobacco. However, the article by Nikogosian and Kickbusch would seem to overlook some of the challenges that may militate against the effective implementation of international law, including the Tobacco Convention, at the national level.

The Framework Convention on Tobacco Control (the Tobacco Convention or Convention) was adopted in 2003.^1^ The Tobacco Convention is one of the most widely ratified international instruments to date. This landmark Convention, which came into force in 2005, has since been ratified by about 180 countries with about 160 signatories.^2^ It was in response to high mortality and morbidity rates associated with smoking across the world. A report has shown that an estimated 6 million people die annually as a result of tobacco.^3^ Although the Tobacco Convention is not a human rights treaty, its proper implementation will go a long way in reducing mortality and morbidity associated with smoking and thus promote the enjoyment of the right to the highest attainable standard of health. Nikogosian and Kickbusch would seem to argue that the adoption of the Convention has led to a significant development at the international level.^4^ According to the authors, the impact of the Convention cannot be underestimated as it has demonstrated how international law may influence public health at the national level.

The authors examine the five potential impacts of the Tobacco Convention and its Protocol on public health. These include, unlocking the treaty-making powers of World Health Organization (WHO); global phenomenal impact of the Convention in international public health architecture; facilitating the adoption of further instruments to strengthen the implementation at the national level; adoption of appropriate legal framework to combat the use of tobacco at the national level and widely recognised impact of the Convention beyond public health to also include the adoption of the Protocol to Eliminate Illicit Trade in Tobacco.

This position of the authors has been corroborated by other authors. For instance, in their study, Gravety et al examine the implementation of the key-demand reduction measures of the WHO Framework on Tobacco Control in 126 countries.^5^ This comprehensive review shows that between 2007 and 2014, there was a significant global increase in highest-level implementation of all key demand-reduction measures of the Tobacco Convention. According to the authors, the proper implementation of the reduction-measures aspect of the WHO Tobacco Convention is associated with lower smoking prevalence with the possibility of future reduction in tobacco related mortality and morbidity. This finding is no doubt significant and would seem to lend credence to the argument of Nikogosian and Kickbusch. It would seem to affirm the concerted commitment of the international community to curbing the negative impact of smoking in the world.

However, despite the applaud accorded the Convention by the authors, there are few issues for consideration. First, the application of international law at the national level remains a subject of controversy. While attempts have been made by some countries to implement the Tobacco Convention at the national level, the degree of success differs. This may be attributed to the differences in socio-economic developments of the countries. Effective implementation of the Convention at the national level requires resources and capacity on the part of states. Given the uneven development between rich and poor countries, the degree of implementation is bound to differ. The report of the Expert Group to assess the impact of the Tobacco Convention at the national level has noted that the implementation of the Convention at the national level has occurred with varied degree of success.^6^ It should be noted that the application of the Convention at the national level, like all other international treaties, will depend on the legal system of a country. While in some countries it may apply directly (monism), in others it may require further actions from the legislature before it can apply (dualism).^7^

Second, while there is good reason to celebrate the positive efforts made so far by states to implement the Tobacco Convention at the national level through adoption of laws and policies, there is need for caution about the effectiveness of these measures. Undoubtedly, adopting laws and policies as required by an international instrument is laudable, however, this is not a guarantee that those laws and policies will be effectively implemented. There is great need for states to exhibit good political will at the national level for the implementation of provisions of the Convention. Experience has shown that even when favourable legal frameworks exist at the national level to address a specific issue, states tend to fare poorly regarding proper implementation of. In *Minister of Health v Treatment Action Campaign,*^8^ a group of civil society organisations led by the Treatment Action Campaign challenged the South African government’s antiretroviral treatment programme, which limited access to life-saving medication to prevent- mother-to-child transmission of HIV to few centres on the basis of a lack of resources and concerns about the efficacy of the medicines. The Court held that the government’s action was unreasonable and amounted to a violation of its obligations under the Constitution and international law to realise the right to health. While this case deals with realising the right to health at the national level, it nonetheless, exemplifies that the existence of a favourable legal framework is no guarantee for effective implementation at the national level. Thus, there is need for constant monitoring of steps and measures adopted by states to effectively implement the Convention at the national level.

Third, the ink of commentators is not dried regarding the weakness of the enforcement mechanisms of international law. Chapman has argued that the Achilles’ heels of international human rights system is the inability to ensure its enforcement against erring states.^9^ Given that states are the primary subject of international law; it is always a challenge to devise a means of dealing with states that fall short of their obligations under ratified treaties. Thus, treaty monitoring bodies or other mechanisms established to ensure implementation of a treaty often lack the wherewithal to compel states to fulfil their obligations when found wanting. At best, what is open to these bodies is to ‘name and shame’ a state that has failed to live up to its obligations to respect, protect and fulfil rights. Although the Tobacco Convention is not a human rights treaty, it is an international instrument and as such is not immune from this challenge. In essence, there is no assurance that states that fail to fully comply with the provisions of the Convention will be sanctioned or caused to toe the line.

Perhaps one of the major concerns regarding the Tobacco Convention relates to the monitoring mechanisms under article 21. By virtue of this provision, a Conference of Parties (COP), made of member states to the Convention, will oversee its implementation at the national level. A state is required to submit its initial report 2 years after ratification of the treaty. Thereafter, the Conference of Parties will determine the periodicity of reports to be submitted by states. The COP plays a key role in monitoring the implementation of the Tobacco Convention. These include, developing guidelines, negotiating Protocols, requesting preparation of technical reports and recommendations, mobilising financial resources and mechanisms of assistance. It has the responsibility of regularly reviewing implementation of the Convention and taking decisions necessary to promote effective implementation, as well as enabling Parties to understand and learn from each other’s experiences.^10^ Since the COP is made of states parties to the Convention, it is paradoxical calling on duty-bearers under the Convention to assume the supervisory role of ensuring the implementation of the same instrument. It is like asking a person to become a judge in his/her own cause. This is contrary to the well-known principle of natural justice that no person should be made to be a judge in his/her own cause-*nemo judex in causa sua*.

One of the challenges with states reports to treaty monitoring bodies is that states are often unfaithful in fulfilling their reporting obligations. This makes it difficult for treaty monitoring bodies to assess the extent to which states have complied with their obligations under a specific treaty. Indeed, evidence from member states of the Tobacco Convention shows some inconsistencies in reporting status. For instance, while South Africa has submitted five reports since ratifying the Convention in 2006, Fiji, which ratified in 2003 and Chad in 2006 have submitted three and two reports respectively. This may tend to undermine the monitoring role of the COP.

Another major gap not peculiar to the Tobacco Convention is the inability to clearly provide for means of holding tobacco companies accountable in implementing the provisions of the Convention. Like most international instruments, the Tobacco Convention is binding on states, which are expected to monitor the activities of tobacco companies that are not parties to the Convention. This has always led to a serious challenge under international law. Indeed, one of the major set-backs of international human rights is its inability to hold non-state actors accountable for human rights violations.^9^ While article 5(3) of the Tobacco Convention provides that states should ensure that the tobacco industry does not interfere with the implementation of the Convention at the national level, there is no clear means of realising this. Therefore, this remains an arduous task for developing countries to achieve. Experience has shown that multinational companies, including tobacco companies, exert great power and influence at the national level and are sometimes difficult to ‘tame’ by the host countries. This may be true of poor countries, where tobacco companies operate, creating employment for a large number of the population and contributing greatly to the income of the host country. In such situations, it may be impracticable for some host countries to fully exercise the nature and degree of control over tobacco companies as envisaged by the Convention. A report by the Expert Group to assess the impact of the Tobacco Convention at the national level shows that the ‘Tobacco industry continues in many ways to resist the FCTC and its Articles, and to oppose, undermine and delay implementation of all measures that may reduce its sales and promotional activities.’^6^ According to the report, this is done through the following means: (*i*) Use of third party or front groups and ‘socially responsible’ activities, (*ii*) Litigation and other legal measure to oppose and delay evidence-based measures, (*iii*) Use of international trade and related agreements to oppose regulatory measure, and (*iv*) Efforts to present the industry as a partner, particularly through new product strategies.^7^ The report therefore recommends for a ‘strong, coordinated and transparent’ application of article 5 (3).

Recent developments have shown that more concerted efforts are being made by the international community to hold non-state actors accountable for human rights violations. The adoption by the Human Right Council of the Guiding Principles on Business and Human Rights^11^ and guidelines to ensure corporate responsibility on the part of pharmaceutical companies are just few of these initiatives.^12^ As part of these initiatives, non-state actors are expected to exhibit high degree of corporate responsibility within the host country. Moreover, non-state actors are expected to conduct themselves in such a way that their actions or omissions will not lead to harm in the host country.^12^ For tobacco companies, this would imply that they are to ensure that their activities will not constitute harm to the host country. It is hoped that these initiatives will lead to a situation where non-state actors will become more accountable to respecting, protecting and fulfilling of human rights.

## Conclusion

The Tobacco Convention is undoubtedly a landmark development of the 21st century. Apart from being the first WHO-led initiative that culminated in a binding instrument, the speed at which it came into force and the high number of ratifications point to the importance of this instrument. Moreover, since its entry into force, the world has witnessed more determined and committed efforts on the part of states to ensure implementation of the provisions of the Tobacco Convention. Much as these developments should be commended, we should not be deluded about the challenges that may hinder the effective implementation of the Convention at the national level. Moreover, the Convention like all international instruments is not insulated from the controversies and challenges that often make the application of international law difficult at the national level.

## Ethical issues

Not applicable.

## Competing interests

Author declares that he has no competing interests.

## Author’s contribution

ED is the single author of the paper.
